# Fast surveillance response reveals the introduction of a new yellow fever virus sub-lineage in 2021, in Minas Gerais, Brazil

**DOI:** 10.1590/0074-02760220127

**Published:** 2022-12-02

**Authors:** Miguel Souza Andrade, Fabrício Souza Campos, Cirilo Henrique de Oliveira, Ramon Silva Oliveira, Aline Alves Scarpellini Campos, Marco Antônio Barreto de Almeida, Vagner de Souza Fonseca, Danilo Simonini-Teixeira, Anaiá da Paixão Sevá, Andrea Oliveira Dias Temponi, Fernando Maria Magalhães, Danielle Costa Capistrano Chaves, Maira Alves Pereira, Ludmila Oliveira Lamounier, Givaldo Gomes de Menezes, Sandy Micaele Aquino-Teixeira, Maria Eduarda Gonçalves-dos-Santos, Sofía Bernal-Valle, Nicolas Felipe Drumm Müller, Jader da Cruz Cardoso, Edmilson dos Santos, Maria Angélica Mares-Guia, George Rêgo Albuquerque, Alessandro Pecego Martins Romano, Ana Cláudia Franco, Bergmann Morais Ribeiro, Paulo Michel Roehe, Filipe Vieira Santos de Abreu

**Affiliations:** 1 Universidade de Brasília, Instituto de Ciências Biológicas, Departamento de Biologia Celular, Laboratório de Baculovírus, Brasília, DF, Brasil; 2 Universidade Federal do Tocantins, Laboratório de Bioinformática e Biotecnologia, Gurupi, TO, Brasil; 3 Universidade Federal do Rio Grande do Sul, Instituto de Ciências Básicas da Saúde, Porto Alegre, RS, Brasil; 4 Instituto Federal do Norte de Minas Gerais, Laboratório de Comportamento de Insetos, Salinas, MG, Brasil; 5 Secretaria Estadual de Saúde do Rio Grande do Sul, Centro Estadual de Vigilância em Saúde, Porto Alegre, RS, Brasil; 6 Organização Pan-Americana da Saúde/Organização Mundial da Saúde, Brasília, DF, Brasil; 7 Stellenbosch University, School of Data Science and Computational Thinking, Centre for Epidemic Response and Innovation, Stellenbosch, South Africa; 8 Universidade Estadual de Santa Cruz, Departamento de Agricultura e Ciências Ambientais, Ilhéus, BA, Brasil; 9 Secretaria de Saúde do Estado de Minas Gerais, Coordenação Estadual de Vigilância de Arbovírus, Belo Horizonte, MG, Brasil; 10 Fundação Ezequiel Dias, Laboratório Central de Saúde Pública, Belo Horizonte, MG, Brasil; 11 Fundação Oswaldo Cruz-Fiocruz, Instituto Oswaldo Cruz, Laboratório de Flavivírus, Rio de Janeiro, RJ, Brasil; 12 Ministério da Saúde, Coordenação Geral de Vigilância de Arbovírus, Brasília, DF, Brasil

**Keywords:** yellow fever virus, Arbovirus, Flavivirus, non-human primate, epizootic, smartphone, MinION

## Abstract

**BACKGROUND:**

In Brazil, the yellow fever virus (YFV) is maintained in a sylvatic cycle involving wild mosquitoes and non-human primates (NHPs). The virus is endemic to the Amazon region; however, waves of epidemic expansion reaching other Brazilian states sporadically occur, eventually causing spillovers to humans.

**OBJECTIVES:**

To report a surveillance effort that led to the first confirmation of YFV in NHPs in the state of Minas Gerais (MG), Southeast region, in 2021.

**METHODS:**

A surveillance network was created, encompassing the technology of smartphone applications and coordinated actions of several research institutions and health services to monitor and investigate NHP epizootics.

**FINDINGS:**

When alerts were spread through the network, samples from NHPs were collected and YFV infection confirmed by reverse transcription-quantitative polymerase chain reaction (RT-qPCR) and genome sequencing at an interval of only 10 days. Near-complete genomes were generated using the Nanopore MinION sequencer. Phylogenetic analysis indicated that viral genomes were related to the South American genotype I, clustering with a genome detected in the Amazon region (state of Pará) in 2017, named YFV_PA/MG_ sub-lineage. Fast YFV confirmation potentialised vaccination campaigns.

**MAIN CONCLUSIONS:**

A new YFV introduction was detected in MG 6 years after the beginning of the major outbreak reported in the state (2015-2018). The YFV strain was not related to the sub-lineages previously reported in MG. No human cases have been reported, suggesting the importance of coordinated surveillance of NHPs using available technologies and supporting laboratories to ensure a quick response and implementation of contingency measures to avoid YFV spillover to humans.

In Brazil, the yellow fever virus (YFV) (family *Flaviviridae*, genus *Flavivirus*) exhibited two epidemiologically distinct transmission cycles: urban and sylvatic. In the urban cycle, which has not been recorded in Brazil since 1942, the virus is transmitted among humans by the vector *Aedes aegypti*.^1^ ^,^ ^2^ In the sylvatic/jungle cycle, the virus is transmitted by wild mosquitoes (mainly belonging to *Haemagogus* and *Sabethes* genera) to non-human primates (NHPs) (e.g., those belonging to *Alouatta* and *Callithrix* genera) and occasionally to unvaccinated humans in close contact with forest areas.^3^ Yellow fever (YF) is endemic to the tropical rainforest of the Amazon region; from this region, waves of epidemic expansion spread toward other Brazilian regions at irregular intervals of time.^3^ During these waves, the virus usually reaches the states of Goiás (GO), in the Central-West region, and Minas Gerais (MG), in the Southeast region. Occasionally, the virus is spread to other southeast states, such as São Paulo (SP) (in 2000, 2008-2009, 2017-2018),^4^ ^-^ ^10^ Rio de Janeiro (RJ) and Espírito Santo (ES) (between 2017 and 2019).^11^ ^-^ ^14^ Even less frequently, YFV can be detected in the southernmost states of the country: Rio Grande do Sul (RS) (2001, 2008-2009, 2020-2021),^15^ ^-^ ^17^ Paraná (PR) and Santa Catarina (SC) (2018-2020).^18^ The YFV spread outside endemic areas raises at least three main concerns: 1) the possibility of re-emergence of an urban cycle; 2) the increased risk of YF in humans due to heterogeneous vaccination coverage outside the Amazon region; and 3) the extinction of threatened NHP species - especially the genus *Alouatta*, highly susceptible to YFV.^5^ ^,^ ^7^ ^,^ ^19^ ^-^ ^23^

During sporadic expansion waves, MG has been an important corridor through which the virus travels before spreading to other Brazilian states.^14^ In recent times, major outbreaks occurred in MG in 2000-2003, 2010 and 2015-2018 (Fig. 1), with the latter being the largest sylvatic outbreak reported in the last 80 years.^6^ ^,^ ^8^ ^,^ ^24^ ^-^ ^28^ In the 2015-2018 outbreak, two viral sub-lineages were detected, crossing MG by different paths: the YFV_MG/SP/RS_ sub-lineage travelled through the west and southwest of the state, before reaching SP; the YFV_MG/ES/RJ/BA_ sub-lineage spread from the northwest, north, northeast and east of the state, before reaching ES and the state of Bahia (BA).^14^ ^,^ ^17^ ^,^ ^28^ Although epizootics have been reported since the end of 2020 (unfortunately, when no sampling was performed), the last reported viral detection in MG occurred in the first quarter of 2018.^14^ ^,^ ^29^ Nevertheless, the virus was detected in the states of Pará (PA), Tocantins (TO), GO, SP, PR, SC and RS from the end of 2020 to June 2022 (Supplementary Fig. 1),^18^ ^,^ ^30^ ^,^ ^31^ raising awareness of the possibility of reintroduction in MG. 

**Fig. 1:**
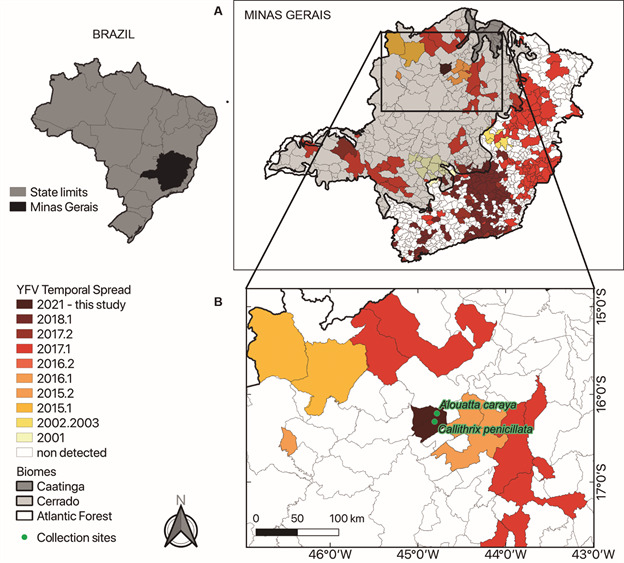
on the left, Brazil map highlighting the state of Minas Gerais (MG). The Brazilian regional division consists of states and municipalities grouped into regions. MG map showing different municipalities with yellow fever virus (YFV) detections in non-human primates (NHPs) and/or humans per semester and year between 2001 to present (see the caption on the left). The different biomes in MG (Caatinga, Cerrado and Atlantic Forest) are shown in gray (A). Location of epizootics in MG registered on the Sistema de Informação em Saúde Silvestre Georreferenciado (SISS-Geo) platform during this investigation (B).

In this work, we describe a surveillance effort encompassing the technology of smartphone applications and the concerted action of Coordenação-Geral de Vigilância Arboviroses, Secretaria de Estado de Saúde de Minas Gerais, Laboratório Central de Saude Pública, Brazilian Ministry of Health, and members of Febre Amarela BR project (Portuguese for Yellow Fever BR) that succeeded in the first YFV confirmation in NHPs in MG in 2021, followed by genome sequencing, in only 10 days. Phylogenetic analyses revealed the introduction of a new sub-lineage in the extra-Amazonian region.

## MATERIALS AND METHODS

*Study area -* This study was conducted in MG, which has the largest number of municipalities (853) in Brazil, the second largest population in the country (21,411,923 inhabitants, 10.1% of the Brazilian population) and the fourth largest area (586,513.99 km^2^, larger than Spain, for example) (https://www.ibge.gov.br/cidades-e-estados/mg.html). The study took place in the north of MG, which is predominantly covered by the Cerrado (a savannah-like biome) (Fig. 1). This region has well-defined dry and rainy periods. August, the month of this investigation, is the driest month of the year, during winter. December presents the highest average rainfall, during summer.

*Establishing an information network to strengthen epizootic surveillance in northern MG -* In 2020, a project sponsored by Conselho Nacional de Desenvolvimento Científico e Tecnológico (CNPq) led to the formation of a network that aggregated research institutions and public health services, aimed at strengthening the surveillance of epizootics in several Brazilian regions.^32^ ^,^ ^33^

**Fig. 2:**
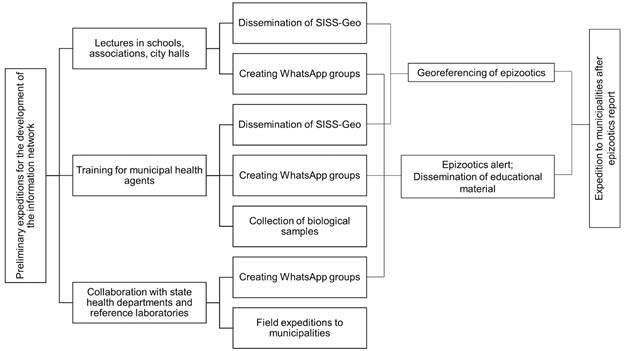
organization chart showing steps for organising the information network to strengthen yellow fever surveillance. SISS-Geo: Sistema de Informação em Saúde Silvestre Georreferenciado.

For these purposes, preliminary field expeditions to 12 municipalities in MG were conducted. In each municipality, meetings and lectures were organised with public health officials and environmental surveillance officials to educate them on the importance of epizootic surveillance as a prompt sign of YFV in circulation. The agents were trained to notify epizootics using the Sistema de Informação em Saúde Silvestre Georreferenciado (SISS-Geo) web app^34^ (or georeferenced wildlife health information system) developed by Fundação Oswaldo Cruz-Fiocruz, attached to the Brazilian Ministry of Health. This platform works as a public repository for collaborative monitoring and disease surveillance of wild animals in Brazil. For monitoring, any person can send pictures of sylvatic animals and mortality (epizootic) in wildlife, as well as metadata. The app collects real-time geographic coordinates and sends information to state and federal surveillance centres. We also organised fieldwork to train teams on vector and NHP sample collection. Finally, several WhatsApp application groups were created with all trainees, constituting an information network whose main aim was to distribute educational material and exchange news about epizootics.^32^ ^,^ ^33^ An organisation chart showing the steps involved in organising the network is shown in Fig. 2. The Secretaria Estadual de Saúde supported all the field expeditions.

*Investigation of the epizootics in northern MG -* On August 24, 2021, the information network alerted us about the occurrence of epizootics in two municipalities, Icaraí de Minas and Ubaí, located in the northern region of MG, 595 km from Belo Horizonte, the capital of the state. Both municipalities remained unaffected during the 2015-2018 outbreak. On August 25, 2021, a team comprising members of the Febre Amarela BR project, members of the Secretaria Estadual de Saúde and officials from the affected municipalities were rapidly assembled and dispatched. On August 25 and 26, 2021, the team surveyed the riparian forests of those municipalities, searching for NHP carcasses to collect biological samples. All detected epizootics were registered in the SISS-Geo app and the Sistema de Informação de Agravos de Notificação (SINAN). Samples were collected following safety protocols,^35^ preserved in liquid nitrogen (-196ºC) and sent to the reference laboratory of the Sistema Único de Saúde (SUS) located at the Fundação Ezequiel Dias (FUNED) in MG, as well as to the laboratory of Febre Amarela BR project for viral diagnosis.

*YFV detection -* Samples (different fragments of viscera) were lysed with TRIzol™, stored in liquid nitrogen and sent to the sequencing laboratory on dry ice (-80ºC). Total RNA extraction with TRIzol was performed according to the manufacturer’s instructions. YFV RNA was detected using a previously published by reverse transcription-quantitative polymerase chain reaction (RT-qPCR) protocol.^36^

*Genome sequencing -* Samples were subjected to cDNA synthesis using the LunaScript™ RT Super-Mix Kit (NEB) following the manufacturer’s instructions. Then, multiplex tiling PCR was performed using previously published YFV primers^37^ with 40 amplification cycles (denaturation: 95°C for 15 s and annealing/extension: 65°C for 5 min) of PCR using Q5 high-fidelity DNA polymerase (NEB). A volume of 7.5 µL of the PCR product was used for DNA library preparation using the Rapid Barcoding Kit 96 SQK-RBK110.96 (Oxford Nanopore Technologies, Oxford, UK) following the manufacturer’s instructions. The sequencing library was loaded onto an R9.4 flow cell (Oxford Nanopore Technologies) and sequenced for 6-18 h using the MiNKOW software. The resulting Fast5 files were base called and demultiplexed using Guppy (Version 4.4.2, Oxford Nanopore Technologies). Consensus sequences were generated by *de novo* assembly using Genome Detective.^38^

*Phylogenetic analyses -* To perform phylogenetic analyses, we selected all near-complete YFV sequences of South American genotype I, available at National Center for Biotechnology Information *(*NCBI) (n = 315), excluding sequences < 8 kb and those of vaccine and patent-related viruses. Metadata such as sample collection dates and geographic positioning were retrieved from GenBank files or genome-associated publications (manual curation). The genome named MG66-L (reported here; details below) and 315 genomes from NCBI were aligned using MAFFT v.7.480.^39^ The subsequent alignment was then used to infer a maximum likelihood tree topology in IQTREE version 2,^40^ using GTR with 1,000 bootstrap replicates. The transfer bootstrap support for splits in the topology was inferred using Booster.^41^ ^)^ The new genome sequences were sent to the NCBI GenBank database under accession numbers OL519587 to OL519589.

## RESULTS

On August 25, 2021 (day 1), following the alert of our information network, members of the Febre Amarela BR project found and notified an epizootic affecting six black-and-gold howler monkeys (*Alouatta caraya*) in Icaraí de Minas (MG), of which only one carcass was suitable for sampling (liver, spleen and kidney samples were collected and named MG66-L, MG66-S and MG66-K, respectively). On August 26 (day 2), another epizootic was detected, affecting six black-tufted marmosets (*Callithrix penicillata*) in Ubaí, and only one carcass met the conditions for sampling collection (from the liver, spleen, kidney and brain, named MG67-L, MG67-S, MG67-k and MG67-B, respectively). Samples were sent to the laboratory (September 2, day 9) and tested (September 3, day 10). All sampled tissues were positive for YFV by RT-qPCR (Table).

**TABLE:** Reverse transcription-quantitative polymerase chain reaction (RT-qPCR) test results, according to tested species, local, tissues, date and Ct

Host species	Municipality	Geographical coordinates	Sample	Collection date	Ct
*Alouatta caraya* (MG66)	Icaraí de Minas	16°13’03.3”S 44°47’00.9”W	Liver	August 25, 2021	15
			Spleen	August 25, 2021	14
			Kidney	August 25, 2021	20
*Callithrix penicillata* (MG67)	Ubaí	16°18’42.0”S 44°48’36.0”W	Liver	August 26, 2021	31
			Spleen	August 26, 2021	30
			Kidney	August 26, 2021	31
			Brain	August 26, 2021	29

**Fig. 3:**
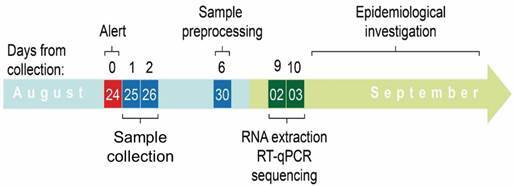
timeline showing the time spent from the collection of samples to the generation of near-complete genomes. RT-qPCR: reverse transcription-quantitative polymerase chain reaction.

The interval between the alert of the information network and YFV diagnosis was 10 days (Fig. 3). After confirming the virus detection, health authorities were immediately notified. Control measures were intensified, and further investigations were conducted in the affected and surrounding areas. Following diagnosis, a sequencing library was prepared, and sequencing was performed on the same day. There was only an 8-hour interval between RNA extraction and generation of the near-whole genome sequence.

Three near-complete YFV genomes were generated from three different fragments of viscera (liver, spleen and kidney) from the same carcass of *Alouatta caraya* (named MG66). A comparison of the three YFV genomes revealed no intra-host sequence differences. Therefore, the MG66-L genome was used for the phylogenetic analysis.

**Fig. 4:**
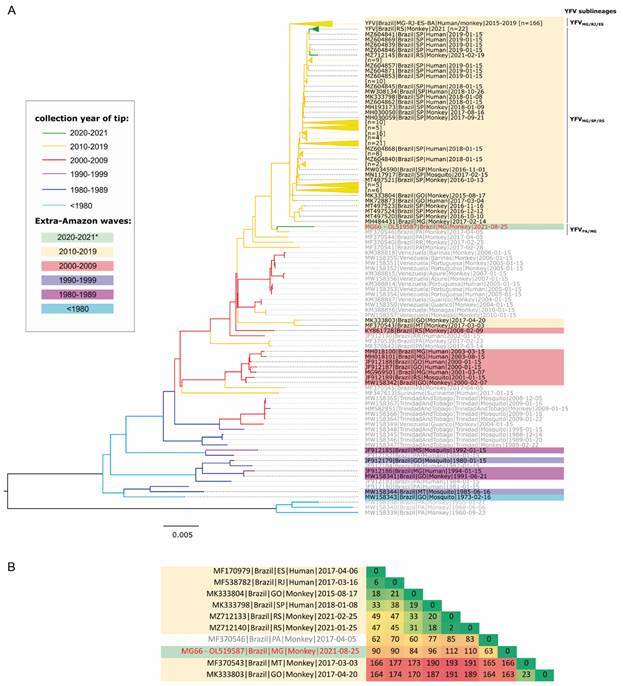
maximum likelihood phylogenetic tree of South American genotype I yellow fever virus (YFV) strains, highlighting in red the MG66-L strain isolated in the southeastern state of Minas Gerais. Branches colours indicate the year of sample collection. Isolates from Amazon region are labelled in gray and isolates from Extra-Amazon region are labelled in black and highlighted by decade of wave (A). The sub-lineage YFV_MG/ES/RJ_ is collapsed at the top of the tree. Nucleotide differences between selected genomes (B).

Such analyses revealed that the YFV genome generated here (MG66 - OL519587|Brazil|MG|Monkey|2021-08-25) was related to the South American genotype I, as expected, and clustered with an isolate (MF370546|Brazil|PA|Monkey|2017-04-05) sampled from an *Alouatta caraya* identified in PA (Amazonian region) in 2017 (Fig. 4A and Supplementary Fig. 2), indicating the introduction of a new YFV sub-lineage in MG, now called YFV_PA/MG_. Moreover, they were not related to the viruses detected in the same year (2021) in RS, the southernmost state of Brazil. Further analysis of the polyprotein encoded by genomes MF370546 (PA-2017) and OL519587 (MG-2021) revealed only five amino acid differences (T128I, X108I, N808D, V3060I and A3149V), whereas comparison of sequences OL519587 and MZ712133 (RS-2021) revealed nine amino acid differences. These findings confirm the expansion and circulation of at least two YFV sub-lineages (YFV_PA/MG_ and YFV_MG/SP/RS_) in the extra-Amazon region in 2021.^17^ The nucleotide difference matrix between the selected genomes is shown in Fig. 4B. 

## DISCUSSION

MG has historically been affected by several YFV introductions. However, owing to limited logistics, it has been difficult to conduct surveillance and collect biological samples promptly, both of which require a robust field and laboratory surveillance infrastructure. In this study, we describe the results of setting up an inter-institutional network capable of collaborative actions to investigate epizootics, which resulted in the first confirmation of YFV circulation in MG, Brazil, in 2021.

MG has played a role as an YFV dissemination route during expansion waves from the Amazon region. Despite the limited resources available in the past, studies on viral dispersion routes in the region have been conducted since 1938 (Supplementary Fig. 3).^24^ ^,^ ^42^ In the last 2 decades, outbreaks that resulted in human cases were recorded in 2001 (32 human cases), 2002-2003 (63 human cases) and two isolated cases between 2008-2009. The largest outbreak occurred between 2016-2018, with 1,006 human cases and 448 confirmed epizootic cases.^26^ ^,^ ^27^ ^,^ ^43^ ^,^ ^44^ Importantly, there were no previous records of viral circulation in the two affected municipalities, suggesting the presence of a naive NHP population.^45^ ^,^ ^46^

Since 2019, to the best of our knowledge, despite the notification of several epizootics, no new reports on YFV circulation have been published in the north of MG.^29^ The wide territorial extension, with huge rural areas far from urban and research centres, combined with socioeconomic inequality and the lack of trained professionals in several municipalities, made it difficult to implement appropriate surveillance of epizootics, thus significantly reducing the chances of timely sample collection for virus detection. These challenges reaffirm the need to introduce new tools and strategies to strengthen surveillance. Among them, the following points are worth mentioning. a) The use of smartphone applications to notify epizootics and collect the geographic coordinates of the occurrence in real time. In this sense, the SISS-Geo app, which has been progressively implemented in Brazil, speeds up the arrival of information to involved institutions and provides accurate geographic location and streamlines outbreak response.^34^ b) The use of instant messaging apps increases the number of “watchers,” providing information through real-time text transmission. Creating WhatsApp groups with a local or regional reach, comprising different members of the population (health agents, cyclists, hikers, students, rural workers, etc.), increases the chances of detecting epizootics and speeding up the dissemination of important information such as vaccination campaigns, NHPs and ecosystem conservation efforts.^32^ ^,^ ^33^ Ultimately, this network of professional and citizen-driven wildlife disease surveillance serves both public health and science popularisation, the latter being a crucial yet neglected part in epidemic management. c) The creation of multi-institutional networks to facilitate and speed up sample collection and diagnosis. In the present study, research institutions linked to the Febre Amarela BR project provided an integrated, multidisciplinary team, aggregating staff from universities, students and governmental organs at municipal, state and federal health secretariats whose coordinates and combined actions allowed the achievement of the results reported here. 

Genomic surveillance efforts have shown that the virus circulating in MG was related to the virus circulating in PA, in the Amazon region, in 2017 (sub-lineage YFV_PA/MG_). This reveals a new wave of viral expansion in MG, 6 years after the outbreak associated with the YFV_MG/SP/RS_ and YFV_MG/ES/RJ/BA_ sub-lineages, between 2015 and 2018. Interestingly, in 2021, at least two different viral sub-lineages were found circulating at the same time in the extra-Amazon region, represented by YFV_MG/SP/RS_, detected in RS,^17^ and YFV_PA/MG_. Notably, the latter was detected at the height of the dry season in one of the driest regions of MG, which makes it more difficult for the main species of mosquito vectors (i.e., *Haemagogus janthinomys* and *Hg. leucocelaenus*) to survive.^13^

In this study, the importance of coordinated efforts between local populations and different institutions is emphasised to increase surveillance capacity. Through this system, it was possible to confirm YFV occurrence, which enabled the establishment of timely response measures. Genomic surveillance has allowed the identification of a new YFV expansion wave in MG, which needs to be monitored to ensure that effective preventive measures are implemented in appropriate time and place. These include an increase in vaccine coverage in humans and improved communication regarding the risk of YFV to health professionals and the general population to avoid human cases. This study also highlights the need for awareness and public information on the role of NHPs as sentinels for the occurrence of YFV. Finally, this study highlights the importance of collaborative, integrated surveillance, allowing prompt action in the event of YF suspicion.
