# Supplementary material for: Fast surveillance response reveals the introduction of a new yellow fever virus sub-lineage in 2021, in Minas Gerais, Brazil
Source: Mem Inst Oswaldo Cruz. 2022 Dec 2;117:e220127. doi: 10.1590/0074-02760220127 (PMC9718055; doi:10.1590/0074-02760220127)
Supplement: Supplementary file 1 [file 1678-8060-mioc-117-e220127-s.pdf]

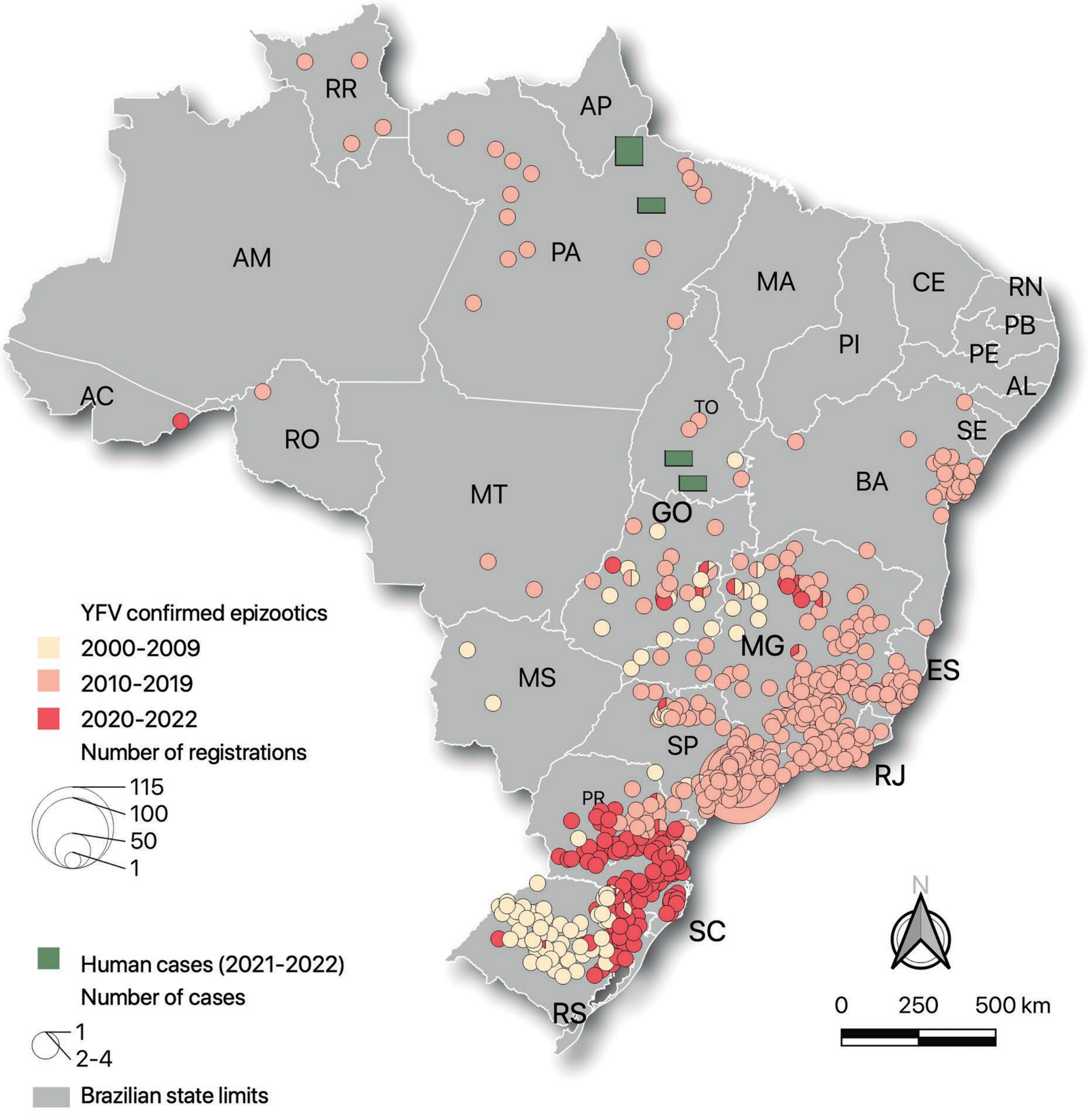

Fig. 1: geographic localization of confirmed yellow fever virus (YFV) detection since 2000. Two main clusters can be observed between July 2020 and June 2022 (red dots), one in the states in Southern Brazil [Paraná (PR), Santa Catarina (SC) and Rio Grande do Sul (RS)] and another in the centre-western states [Goiás (GO) and Distrito Federal (DF)] and southeastern states [Minas Gerais (MG) and São Paulo (SP)]. The yellow fever detection in GO and TO, close to MG, illustrate the transmission route before virus spread to MG, corroborating our findings. Importantly, human cases were also detected in the Brazilian northern region (main PA and TO) in the last 2 years. Source: map adapted from Fig. 3[18] and Fig. 12.[30] We also use data from DataSUS.[31]

CC BY

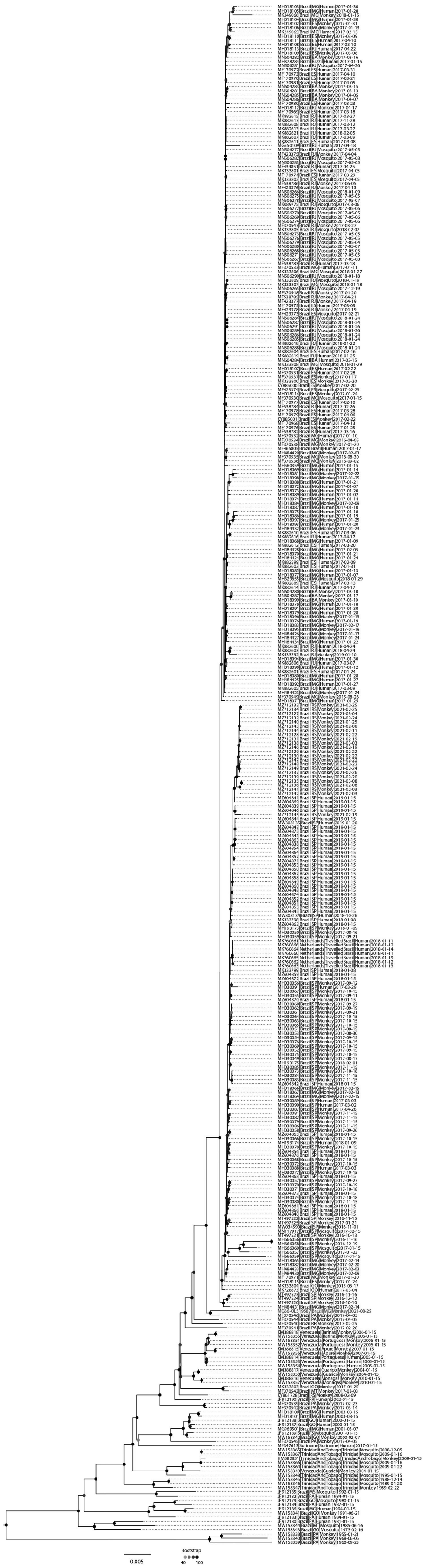

Fig. 2: complete maximum likelihood phylogenetic tree of South American genotype I yellow fever virus (YFV) strains, highlighting in red the MG66-L strain isolated in the southeastern state of Minas Gerais. Bootstrap support values are indicated by circles on nodes according to the legend.

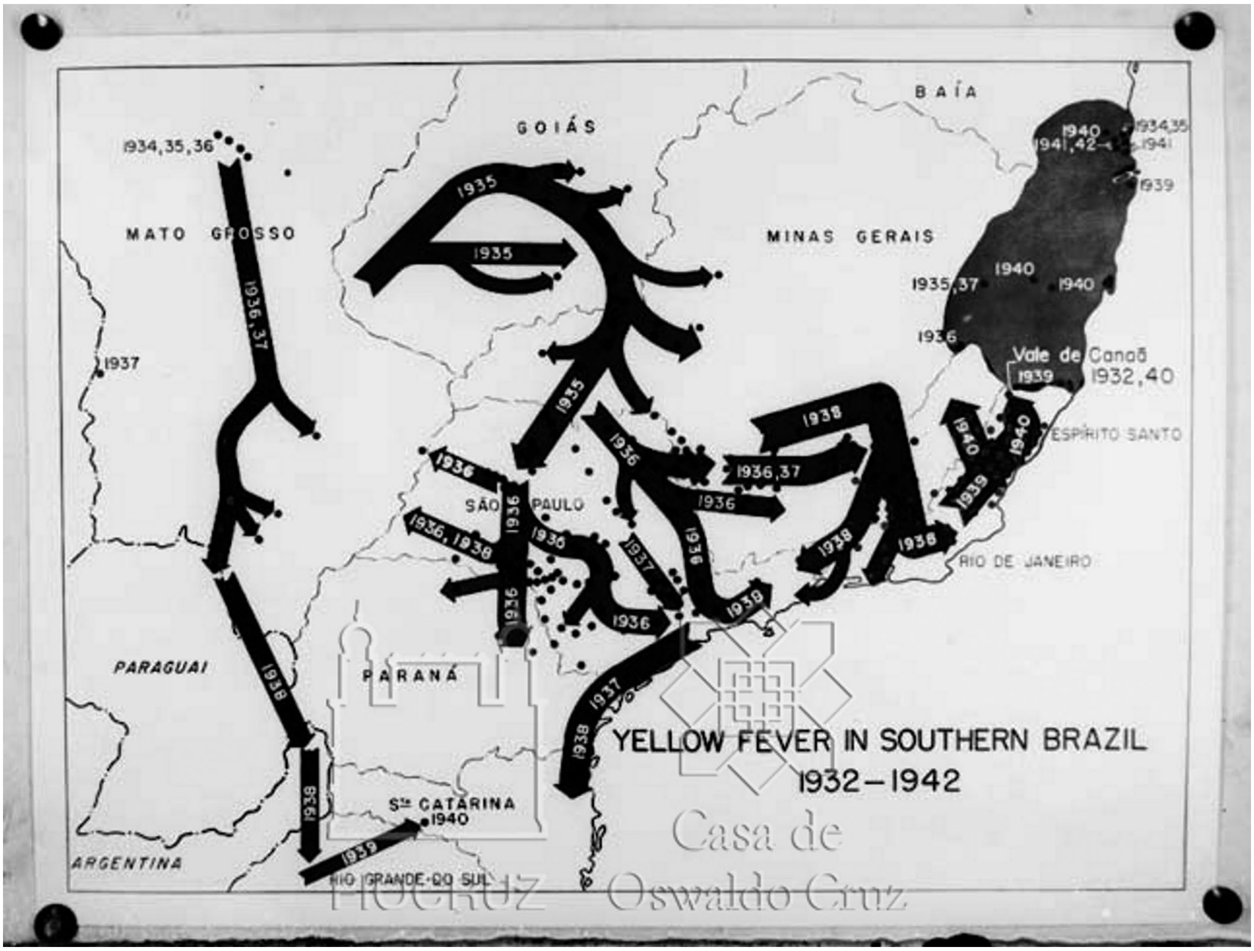

Fig. 3: likely routes of dissemination of yellow fever in southeastern Brazil, 1932-1942. Source: Casa de Oswaldo Cruz.[42]
